# Rapid evolution of cancer/testis genes on the X chromosome

**DOI:** 10.1186/1471-2164-8-129

**Published:** 2007-05-23

**Authors:** Brian J Stevenson, Christian Iseli, Sumir Panji, Monique Zahn-Zabal, Winston Hide, Lloyd J Old, Andrew J Simpson, C Victor Jongeneel

**Affiliations:** 1 Ludwig Institute for Cancer Research and Swiss Institute of Bioinformatics, CH-1015 Lausanne, Switzerland; 2 South African National Bioinformatics Institute, University of the Western Cape, Bellville, 7535, South Africa; 3 Ludwig Institute for Cancer Research, New York Branch at Memorial Sloan-Kettering Cancer Center, 1275 York Avenue, New York, New York 10021, USA

## Abstract

**Background:**

Cancer/testis (CT) genes are normally expressed only in germ cells, but can be activated in the cancer state. This unusual property, together with the finding that many CT proteins elicit an antigenic response in cancer patients, has established a role for this class of genes as targets in immunotherapy regimes. Many families of CT genes have been identified in the human genome, but their biological function for the most part remains unclear. While it has been shown that some CT genes are under diversifying selection, this question has not been addressed before for the class as a whole.

**Results:**

To shed more light on this interesting group of genes, we exploited the generation of a draft chimpanzee (Pan troglodytes) genomic sequence to examine CT genes in an organism that is closely related to human, and generated a high-quality, manually curated set of human:chimpanzee CT gene alignments. We find that the chimpanzee genome contains homologues to most of the human CT families, and that the genes are located on the same chromosome and at a similar copy number to those in human. Comparison of putative human:chimpanzee orthologues indicates that CT genes located on chromosome X are diverging faster and are undergoing stronger diversifying selection than those on the autosomes or than a set of control genes on either chromosome X or autosomes.

**Conclusion:**

Given their high level of diversifying selection, we suggest that CT genes are primarily responsible for the observed rapid evolution of protein-coding genes on the X chromosome.

## Background

Cancer/testis (CT) genes are a growing family of genes defined by a unique pattern of expression: amongst normal tissues, they are expressed only in cells of the germ line and in embryonic trophoblasts, but their gene products are also found in a significant number of malignant cancers [1]. The first CT genes were discovered because of the immune responses that they elicit in some cancer patients, and can thus be classified as CT antigens [2,3]; systematic exploration of publicly available gene expression profiles (as documented in EST libraries, SAGE and MPSS data, and microarray experiments) uncovered a significant number of additional CT genes [4,5], against most of which immune responses have not yet been documented. Nevertheless, all CT genes are in principle attractive targets for cancer immunotherapy, because the gonads are immunoprivileged organs and anti-CT immune responses will therefore target tumours specifically. Vaccination using peptides derived from the NY-ESO-1 (*CTAG1B*) and *MAGEA1 *CT genes has already been proven to bring clinical benefits to melanoma patients [6,7].

CT genes comprise more than 240 members from 70 families, and can be subdivided into two broad categories based on chromosomal localization. CT-X genes are located on the X chromosome, are mostly members of gene families organized into complex direct and inverted repeats, and are expressed primarily during the spermatogonial stage of spermatogenesis [8]. Non-X CT genes are located on autosomes, are mostly single-copy genes, and are expressed primarily during the meiotic and reduction division stages of spermatogenesis [8]. Careful annotation of the sequence of the human X chromosome has revealed that as many as 10% of all genes present on the chromosome are members of known CT families [9]; further analysis of the expression patterns of genes of unknown function located in repeated regions could even increase this estimate [5]. The biological functions of most CT-X genes have not been characterized in any detail. However, evidence is emerging that the best studied of these, the MAGE genes, can act as signal transducing transcriptional modulators. Moreover, MAGE genes appear to be able to mediate proliferative signals [10-12] and a member of the GAGE family has been shown to repress apoptosis [13], thus directly contributing to the malignant phenotype when aberrantly expressed in cancer. Available data suggest that many CT genes are involved in the re-programming of the transcriptional machinery that occurs during the transition from mitotic to meiotic division during spermatogenesis. It has been suggested that a similar re-programming may be responsible for some of the phenotype of malignant cancer cells [8,14].

There is mounting evidence that the evolutionary history of the human X chromosome is significantly different from that of autosomes. It contains a disproportionate number of tandem and interspersed segmental duplications, both direct and inverted, containing genes with a testis-specific expression pattern including many CT-X genes [9]. These duplications are unstable in the genome, and subject to copy number polymorphisms, both within the human population and between humans and chimpanzees [15,16]. While its overall DNA sequence has diverged significantly less than that of autosomes since speciation of hominoids from chimpanzees [17], a significant proportion of protein-coding genes located on the X chromosome are under higher diversifying (positive) selection than those on autosomes [18]. Genes located on the X chromosome are also the most abundant source of functional retrogenes in the primate lineage, and constitute a reservoir of genetic material for the generation of new genes and functions in this lineage, again with a bias toward testis-specific functions [19,20].

For all of these reasons, it is of interest to trace the evolutionary history of CT genes, and particularly of the CT-X subset, and to measure the selective pressures that act on them. Many of the human CT-X genes do not have easily identifiable orthologues in the mouse, rat or dog genomes, precluding such an analysis among Eutheria using currently available genome data. For example, it has been shown that the large MAGE family of CT-X genes has expanded independently in the primate and rodent lineages [21]. The recent availability of a draft genome for the chimpanzee has made it feasible to study the evolution of the CT genes within the primate lineage. We show here that the CT genes in general and the CT-X genes in particular are under strong diversifying pressure and amongst the fastest-evolving genes in the human genome.

## Results

### Identification of CT gene families in chimpanzee

To date at least seventy CT gene families, many with multiple members, have been identified in human. We took the opportunity afforded by the publication of the initial sequence of the chimpanzee genome [18] to ask whether CT genes were conserved in man's closest evolutionary neighbour. To this end we assembled a list of human transcript sequences representing all CT gene families, and searched for homologous sequences in the human and chimpanzee genomes. We expected that given the relatively short time elapsed since human-chimpanzee divergence (~ 6 million years ago [17]) the human sequences would be able to detect CT gene homologues in the chimpanzee genome. Moreover, since the majority of CT genes isolated thus far were detected and characterized using transcript information via cDNA cloning protocols, performing the same search in human allowed us to identify all CT genes present in the current assembly of the human genome. We implemented a two-stage approach in order to accurately define the structure of each CT gene locus. First, we used MegaBlast [22] to search for regions homologous to the CT transcript sequences. Then we applied the SIBsim4 cDNA to genome alignment program (an improved version of sim4 [23]) to these regions to establish a gene structure from a locus-specific spliced alignment (see Methods). As can be seen in Table 1, almost all human CT families are found in chimpanzee, and the chromosomal locations of the CT genes in chimpanzee correspond to those in human. In terms of copy number, the biggest family, PRAME, is well represented in chimpanzee (37 genes), as are MAGEA (9 genes) CTAGE (15 genes), XAGE (12 genes) and SSX (8 genes). The number of CT genes in each family is probably underestimated because of the relatively low sequence coverage in the current version of the chimpanzee genome assembly. This is especially true for the X chromosome, where the sequence coverage is only about 2-fold [18], and where most of the human multi-gene CT families are located. Nevertheless, the current data indicate that some chimpanzee CT families (FTHL17/CT38, TSPY/CT78 and PRAME) may contain more members than in human.

In order to investigate more closely the relatedness of CT genes in these two species, we sought putative human and chimpanzee orthologues for as many CT genes as possible, based on nucleotide sequence identity to the cognate human transcript sequence. Ninety-eight orthologous CT pairs were defined in this way (see Methods and additional file 1). The average identity of the human and chimpanzee orthologues to the human transcript sequences was 99.6% and 97.8%, respectively. Since we were interested in the characteristics of CT genes as a group, we also defined a group of human-chimpanzee orthologous non-CT control genes from chromosome X, where most of the CT genes are located, and from autosomal chromosomes 18 and 19 (see Methods). The reasons for choosing a limited set of control genes were two-fold: first, this allowed us to generate manually curated alignments of the same quality as for the CT genes, and second, it provided test and control groups of similar sizes for statistical analysis. The average identity of the human and chimpanzee control orthologues to the human transcript sequences was 99.6% and 98.7%, respectively. The finding that the chimpanzee and human CT orthologues were on average less closely related than the control orthologues (97.8% versus 98.7%; p < 2.2e-16 by a chi-squared test) suggested a possible difference in the divergence rates between the CT group and the control group. We tested this by analysing the substitution rates between human and chimpanzee ORF sequences (see below). Given the high accuracy of the human genomic sequence, the finding that the average human identity was less than 100% for both CT genes and non-CT control genes presumably reflects polymorphisms and/or sequencing errors in the original transcript sequences.

### CT genes on chromosome X are evolving faster than those on other chromosomes

**Figure 1:**
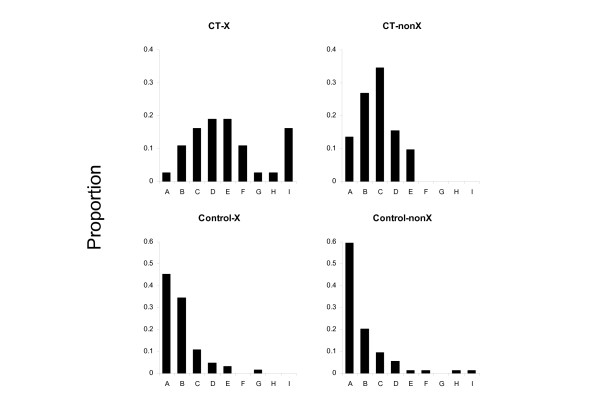
**Distribution of dN/dS ratios for CT genes and controls**. The proportion of genes in each category with ratios in intervals A-I is shown. The categories are: CT-X, CT genes on chromosome X (N = 33); CT-nonX, CT genes not on chromosome X (N = 49); Control-X, control genes on chromosome X (N = 64); Control-nonX, control genes not on chromosome X (N = 71). The intervals are: 0 ≤ A ≤ 0.25; 0.25 < B ≤ 0.5; 0.5 < C ≤ 0.75; 0.75 < D ≤ 1.0; 1.0 < E ≤ 1.25; 1.25 < F ≤ 1.5; 1.5 < G ≤ 1.75; 1.75 < H ≤ 2; 2 < I ≤ 4. Genes which had no synonymous changes (dN/dS denoted '∞' in Table 2) were omitted from the analysis.

We estimated the divergence rates of the CT genes from pairwise sequence alignments of the human and chimpanzee orthologues using phylogenetic analysis (PAML package [24]). Mutations in a protein-coding gene can either have no effect (synonymous changes) or alter the sequence of the encoded protein (non-synonymous changes). The rate of synonymous changes (dS) indicates the background mutation frequency, while the ratio of the non-synonymous to synonymous mutation rates (dN/dS) indicates the type of evolutionary pressure acting on the gene. A dN/dS ratio value less than 1 suggests negative or purifying selection, a ratio equal to 1 suggests neutral evolution, and a ratio greater than 1 suggests positive or diversifying selection [25]. To test what type of evolutionary pressure might be acting on the CT genes, we aligned the ORFs in the human-chimpanzee orthologue pairs and used the codeml program from the PAML package [24] to estimate the dN/dS ratios. Again, for comparison purposes, the control genes were subjected to an identical procedure. Figure 1 shows the distribution of dN/dS ratios for the CT genes and controls by chromosomal location. In contrast to the control genes, which show the distribution of ratios expected if most genes are under purifying selection, CT genes located on chromosome X have an excess of ratios greater than one. At the level of individual genes, *SSX1*, *PAGE2B*, *SSX4*, *MAGEB2*, *GAGE4 *and *CPXCR1 *have rate ratios greater than 2, indicative of strong evolutionary selective pressure acting on the gene products (Table 2). CT genes located on chromosomes other than chromosome X (CT-nonX) have a distribution of ratios skewed towards lower values, suggesting that this subgroup is evolving slower than the CT-X genes. In contrast, the majority of control genes, irrespective of chromosomal location, have rate ratios less than 0.5, suggestive of purifying selection. In addition, the nonsynonymous substitution rates for CT genes which had no synonymous changes between human and chimpanzee was on average higher than for the controls (see additional file 2).

The apparent difference between the dN/dS distributions for the CT genes and the controls was assessed for significance using a nonparametric Mann-Whitney test, which indicates whether the medians of the two populations are significantly different. The difference in dN/dS values between all CT genes and all controls is highly significant with a p-value of 1.128e-11 (Table 3). Moreover, the difference between CT genes and the controls is significant whether the CT genes are located on chromosome X (p = 4.686e-10) or not (p = 1.498e-05). The distribution of dN/dS values is also significantly different for CT genes on chromosome X compared to those elsewhere (p = 2.812e-05), suggesting that there is stronger selective pressure on CT genes located on chromosome X. In contrast, there is no significant difference in the distribution of dN/dS ratios between the control genes located on chromosome X or elsewhere (p = 0.4962). Previous work has shown that the protein-coding genes on the hominid X chromosome have a higher average dN/dS value than other chromosomes [18]. Our results suggest that the CT genes contribute strongly to this difference, and thus to the rapid evolution of protein-coding genes on the X chromosome.

## Discussion

Several recent publications have taken advantage of the chimpanzee draft genome to identify genes that are under diversifying selection in the primate lineage ([26] and references therein). Their conclusions were concordant, in that they identified the X chromosome as containing a high number of positively selected genes, they found that positively selected genes are predominantly testis-specific, and that their functions are linked to gametogenesis as well as sensory perception and immunity against invading pathogens. Because most of these studies were performed at the whole genome level, they tended to focus on genes for which orthologues could be easily identified and pairwise alignments of coding regions generated automatically. This may explain why they failed to identify CT genes as a dominant group of positively selected genes. A review of recently published literature confirms that only a limited number of CT genes have been recognised as undergoing positive selection (Table 4). Moreover, a large proportion were identified through investigation of individual CT gene families (SPANX [27] and PRAME [28]). In the present study, we have focused on the comparison between human and chimpanzee CT genes, with an emphasis on generating high-quality manually curated data. This was made necessary by the fact that many CT genes are located within segmental duplications and hence have multiple paralogues, and that we tried to be exhaustive in our analysis of all known CT gene families. Because of the large number of gaps that remain in the current assembly of the chimpanzee genome and the relatively high stringency we imposed on the extent of the alignments, we have certainly underestimated the number of CT homologues present in the chimpanzee genome, and some of the human:chimpanzee pairs may not correspond to true orthologues. However, neither of these problems should significantly affect the main conclusions of our study.

Given the close evolutionary kinship between humans and chimpanzees it is not surprising that all known CT gene families are shared between the two species. On the other hand, homologues of many CT antigens have not been found outside the primate lineage so far, and the available genome data are still too sparse to track the appearance of CT gene families during mammalian evolution. Even though the data are still incomplete, it is clear that most CT gene families are undergoing copy number expansions in the primate lineage, presumably driven by non-allelic homologous recombination between segmental duplications. The best-studied CT family in this respect is SPANX, which is present as a single-copy gene in rodents and has duplicated and acquired new sub-families in the primate lineage, including at least one (*SPANX-C*) found to be specific to humans on the basis of its genomic position [27]. SPANX genes have been shown to have copy number polymorphisms in the human population, potentially linked to susceptibility to prostate cancer, and to undergo very rapid evolution affecting both dN and dS [29]. An elegant study of the PRAME cluster on human chromosome 1 [28] revealed the recent expansion in the human lineage of these genes via two large segmental duplications, and subsequent smaller duplications that may be polymorphic in the human population. The large MAGE family of CT antigens, which also comprises genes that do not show a CT expression pattern, has expanded in both the primate and rodent lineages, but independently [21]. Our data also show that many MAGE genes are under diversifying selection (Table 2).

By definition, CT genes are expressed in testis, and for those for which data exists expression has been shown to be restricted to cells involved in spermatogenesis. It is believed that many CT genes are also expressed during oogenesis, but data on this process are still very sparse [30,31]. There is abundant evidence in the literature that many genes expressed predominantly during gametogenesis, as well as those implicated in reproduction in general (e.g. those encoding proteins found in the seminal fluid or expressed predominantly in the prostate) are undergoing positive selection during evolution [32-34]. In this respect, CT genes seem to behave much like other reproductive genes.

However, the CT-X genes are a special case, in that diversifying selective pressure seems more intense on this class. It is probable that the evolutionary pressures driving changes in the encoded protein sequences and those driving the expansion of the CT-X gene families are similar. Strikingly, the X chromosome is enriched in intrachromosomal tandem segmental duplications relative to autosomes [9]. Several hypotheses have been put forward to explain why a subset of genes located on the X chromosome is evolving faster than those on autosomes [34-36]. Our data do not shed new light on this subject. However, it is interesting to note that CT-X genes contribute very significantly to the high average positive selection observed in protein-encoding genes on this chromosome, against a genomic background that is much more highly conserved than on the autosomes [17]. One may speculate that transcriptional controls on recently duplicated genes could be relaxed relative to the parental copies, thereby allowing re-expression in tumours and the partial replication in these tumours of the transcriptional changes accompanying gametogenesis.

## Conclusions

Essentially all human CT families have homologues at the same chromosomal locations in the chimpanzee genome. The copy numbers in the multi-gene CT families may differ between the two species but until a high-quality assembly of the chimpanzee genome is available this cannot be assessed in a reliable way. On the average, CT genes are under stronger positive selection than a set of randomly selected control genes. CT-X genes as a group are evolving very rapidly, not only relative to control genes on the X chromosome or on autosomes, but also relative to autosomal CT genes.

## Methods

### CT genes and human/chimpanzee genomic sequences

Human Reference sequence (RefSeq [37]), or GenBank (where no RefSeq was available) entries were obtained for transcripts representing all documented CT gene families in the CT Gene Database [38]. Transcript sequences were also obtained for additional candidate CT genes described in recent publications, which have not yet been added to the CT Gene Database. In some cases, multiple alternatively spliced transcript sequences from the same gene were selected to maximize sequence representation of the locus. Although PRAME has not been designated a CT gene, due to its trace level of expression in some normal adult tissues other than testis, it does exhibit the other main characteristics of CT genes, i.e. strong expression in the testis and up-regulation in various tumours, and was included in the set of CT genes selected for this study. Non-CT control genes were randomly chosen from lists of genes having a RefSeq identifier on chromosomes X, 18 (low gene density) and 19 (high gene density), generated using BioMart [39,40]. Control genes were selected from locations distributed uniformly along the lengths of the chromosomes to average out site-specific differences in mutation rates. The human (Homo sapiens) genomic sequence used was NCBI Build Number 36 (version 1, release date 9 March 2006), obtained from the NCBI. The chimpanzee (Pan troglodytes) genomic sequence used was NCBI Build Number 2 (version 1, release date 4 October 2006), also obtained from the NCBI.

### Identification of CT gene loci in human and chimpanzee

CT gene loci were identified in both human and chimpanzee based on sequence identity between the human transcript sequences and human or chimpanzee genomic sequences. We used MegaBlast [22] to identify genomic regions homologous to the RefSeq sequences and SIBsim4 [41] (an improved version of sim4 [23]) to produce high quality spliced alignments at those sites, from which locus-specific transcript sequences were generated. A gene was considered complete if the alignment contained at least 80% of the cognate transcript length or 80% of the annotated open reading frame (ORF), and had at least 85% identity to the human transcript sequence. Putative orthologues were identified as the sequences in human and chimpanzee genomes having the highest identity (and satisfying the 80% length threshold) to the same human transcript sequence. In many cases the poor quality (gaps, incorrect assembly) of the published chimpanzee genome sequence prevented us from finding a chimpanzee orthologue to the human gene. High quality sequence alignments for putative human/chimpanzee orthologues were obtained for 98 of the initial list of 135 CT genes (73%) and 153 of the 180 control genes (85%) selected randomly from chromosomes 18, 19 and X.

### Divergence of CT genes

The genome-based transcript sequences derived from human and chimpanzee for each putative orthologous pair were aligned using clustalw (version 1.81 [42]), with gap extension penalties set to zero to allow gaps in the alignment arising from sequences missing in the chimpanzee assembly. Both sequences in the alignment were then trimmed to the extent of the human ORF based on annotation in the RefSeq or GenBank entry. Each nucleotide alignment was manually curated and revised, if necessary, to reflect the corresponding protein alignment. ORFs containing stop codons were dropped from the analysis. Rates of synonymous (dS; also known as Ks) and non-synonymous (dN; also known as Ka) substitutions between aligned ORFs were estimated using the codeml programme from the PAML package [24] with the F3x4 codon frequency model (and runmode = -2 in the codeml control file). Note that incomplete codons in either the human or the chimpanzee sequence are ignored by codeml. The statistical significance of differences in the distributions between human-chimpanzee divergence rates (dN/dS) among CT genes and controls was assessed using a Mann-Whitney (Table 3) or Welch two sample t-test (additional file 3) in the R package [43].

## Abbreviations

CT – cancer/testis

CT-X – CT genes on chromosome X

dN – nonsynonymous substitution rate

dS – synonymous substitution rate

NCBI – National Center for Biotechnology Information

ORF – open reading frame

PAML – phylogenetic analysis by maximum likelihood

## Authors' contributions

BJS, CI, LJO, AJS and CVJ designed the experiments. BJS wrote the software pipeline to identify human and chimpanzee CT genes and to produce ORF alignments. SP, MZ and WH scanned the literature for citations of positive selection. BJS and CVJ wrote the manuscript, which was read and approved by all authors.

## Supplementary Material

Additional File 1 **Homology data on the human:chimpanzee putative orthologues used in this study**. Excel spreadsheet presenting homology data on the human:chimpanzee putative orthologues. Click here for file

Additional File 2 **Phylogenetic analysis of CT and control gene ORFs using codeml**. Excel spreadsheet presenting data additional to that displayed in Table 2. Click here for file

Additional File 3 **Significance of the differences in the distributions of dN/dS ratios between CT and control ORFs using a parametric t-test**. Distribution of dN/dS ratios assessed by parametric t-test. The results are qualitatively similar to those presented in Table 3 and confirm that the distribution of dN/dS values is different between CT genes and controls. Click here for file

## Figures and Tables

**Table 1:** Number and chromosomal location of CT genes in human and chimpanzee

**CT Number**	**Family Name**	**Human Chromosome**	**Human Gene Number**	**Chimpanzee Chromosome**	**Chimpanzee Gene Number**
CT1	MAGEA	X	13 (0)	X	9 (0)
CT2	BAGE	5, 7, 9, 18, 21	7 (0)	7, 9, 18	4 (0)
CT3	MAGEB	X	7 (1)	X	7 (1)
CT4	GAGE	X	16 (0)	X	3 (0)
CT5	SSX	X	14 (0)	X	8 (0)
CT6	CTAG	X	3 (0)	X	1 (0)
CT7	MAGEC	X	2 (0)	X	1 (0)
CT8	SYCP1	1	1 (0)	1	1 (0)
CT9	BRDT	1	1 (0)	1	1 (0)
CT10	MAGEE	X	2 (2)	X	1 (1)
CT11	SPANX	X	11 (0)	X	4 (0)
CT12	XAGE	X	14 (0)	X	12 (0)
CT13	DDX43	6	1 (0)	6	1 (0)
CT14	SAGE	X	1 (0)	X	1 (0)
CT15	ADAM2	4, 8	2 (0)	4, 8	2 (0)
CT16	PAGE	X	7 (0)	X	6 (0)
CT17	LIPI	21	2 (0)	-	0 (0)
CT21	CTAGE	2, 6, 7, 9, 10, 13, 14, 18	21 (12)	2B, 6, 7, 9, 10, 13, 14, 18	15 (6)
CT24	CSAG	X	4 (0)	X	2 (0)
CT25	DSCR8	21	2 (0)	-	0 (0)
CT26	DDX53	X	1 (1)	X	1 (1)
CT27	CTCFL	20	1 (0)	20	1 (0)
CT28	LUZP4	X	1 (0)	X	1 (0)
CT29	CASC5	15	1 (0)	15	1 (0)
CT30	TFDP3	13, 15, X	4 (3)	15, X	2 (2)
CT32	LDHC	11	1 (0)	11	1 (0)
CT33	MORC1	3	1 (0)	3	1 (0)
CT34	DKKL1	19, 20	2 (1)	19, 20	2 (1)
CT35	SPO11	20	1 (0)	20	1 (0)
CT36	CRISP2	6	1 (0)	6	1 (0)
CT37	FMR1NB	X	1 (0)	X	1 (0)
CT38	FTHL17	X	4 (4)	X	5 (5)
CT39	NXF2	X	2 (0)	X	1 (0)
CT41	TDRD	6, 10	2 (0)	6, 10	2 (0)
CT42	TEX15	8	1 (0)	8	1 (0)
CT43	FATE1	X	1 (0)	X	1 (0)
CT44	TPTE	13, 21, Y	4 (0)	13	1 (0)
CT45	CT45	X	6 (0)	X	4 (0)
CT46	HORMAD1	1, 6	2 (1)	1, 6	2 (1)
CT47	LOC255313	X	12 (0)	X	2 (0)
CT48	SLCO6A1	5	1 (0)	5	1 (0)
CT49	TAG	5	1 (0)	5	1 (0)
CT50	LEMD1	1	1 (0)	1	1 (0)
CT51	HSPB9	17	1 (1)	17	1 (1)
CT53	ZNF165	6	1 (0)	6	1 (0)
CT54	SPACA3	17	1 (0)	-	0 (0)
CT55	CXorf48	X	3 (0)	X	1 (0)
CT56	THEG	19	1 (0)	19	1 (0)
CT57	ACTL8	1	1 (0)	1	1 (0)
CT58	NALP4	19	1 (0)	19	1 (0)
CT59	COX6B2	19	1 (0)	19	1 (0)
CT60	BC047459	15	2 (0)	Un	1 (0)
CT61	CCDC33	15	1 (0)	15	1 (0)
CT62	BC048128	15	1 (0)	15	1 (0)
CT63	PASD1	X	1 (0)	X	1 (0)
CT65	TULP2	19	1 (0)	19	1 (0)
CT66	AA884595	7	1 (1)	7	1 (1)
CT68	MGC27016	4	1 (0)	4	1 (0)
CT69	BC040308	6	1 (0)	6	1 (0)
CT71	SPINLW1	20	1 (0)	20	1 (0)
CT72	TSSK6	19	1 (1)	-	0 (0)
CT73	ADAM29	4	1 (0)	4	1 (0)
CT74	CCDC36	3	1 (0)	3	1 (0)
CT75	BC033986	2	1 (0)	2B	1 (0)
CT76	SYCE1	10	1 (0)	10	1 (0)
CT77	CPXCR1	X	1 (0)	X	1 (1)
CT78	TSPY1	Y	14 (0)	Y	22 (0)
CT79	TSGA	2, 21	3 (0)	2A	1 (0)
CT81	ARMC3	10	1 (0)	10	1 (0)
CTNA	PRAME	1, 22	36 (0)	1, 22, Un	37 (0)

CT gene families are presented in numerical order according to proposed nomenclature [1]. The largest family, PRAME, has not yet been assigned official CT designation. Total gene number for each family was determined according to sequence identity and completeness (see Methods). Numbers in brackets denote the number of intronless gene copies, which in the case of multi-exon genes may indicate putative retrocopy genes.

**Table 2:** Nucleotide substitution rates estimated from alignments of human and chimpanzee orthologous CT ORFs

**Gene Name**	**Refseq**	**Chromosome**	**dN**	**dS**	**dN/dS**
*ACTL8*	NM_030812	1	0.0012	0.0170	0.0700
*BRDT*	NM_207189	1	0.0066	0.0071	0.9216
*HORMAD1*	NM_032132	1	0.0068	0.0104	0.6485
*LEMD1*	NM_001001552	1	0.0044	0.0327	0.1342
*PRAMEF1*	NM_023013	1	0.0162	0.0288	0.5624
*PRAMEF2*	NM_023014	1	0.0304	0.0317	0.9573
*PRAMEF3*	NM_001013692	1	0.0223	0.0269	0.8278
*PRAMEF4*	NM_001009611	1	0.0284	0.0305	0.9314
*PRAMEF5*	NM_001013407	1	0.0353	0.0586	0.6025
*PRAMEF6*	NM_001010889	1	0.0142	0.0149	0.9479
*PRAMEF8*	NM_001012276	1	0.0141	0.0262	0.5383
*PRAMEF10*	NM_001039361	1	0.0184	0.0262	0.7029
*PRAMEF16*	NM_001045480	1	0.0253	0.0236	1.0734
*SYCP1*	NM_003176	1	0.0050	0.0123	0.4093
*BX103208*	BX103208	3	0.0000	0.0346	0.0009
*CCDC36*	NM_178173	3	0.0065	0.0118	0.5502
*MORC1*	NM_014429	3	0.0071	0.0112	0.6325
*CCDC110*	NM_152775	4	0.0081	0.0142	0.5694
*MGC27016*	NM_144979	4	0.0017	0.0166	0.0994
*SLCO6A1*	NM_173488	5	0.0083	0.0093	0.8940
*TAG1*	AY328030	5	0.0001	0.1321	0.0009
*BC040308*	BC040308	6	0.0381	0.0004	∞
*CRISP2*	NM_003296	6	0.0034	0.0078	0.4355
*DDX43*	NM_018665	6	0.0046	0.0084	0.5422
*TDRD6*	NM_001010870	6	0.0029	0.0077	0.3756
*ZNF165*	NM_003447	6	0.0028	0.0083	0.3332
*AA884595*	AA884595	7	0.0000	0.0000	0.4503
*BAGE2*	NM_182482	7	0.0000	0.0000	0.4741
*ADAM2*	NM_001464	8	0.0090	0.0102	0.8787
*TEX15*	NM_031271	8	0.0064	0.0103	0.6188
*BAGE*	NM_001187	9	0.0000	0.0441	0.0009
*ARMC3*	NM_173081	10	0.0049	0.0142	0.3479
*SYCE1*	NM_130784	10	0.0073	0.0105	0.6979
*TDRD1*	NM_198795	10	0.0035	0.0085	0.4101
*LDHC*	NM_002301	11	0.0000	0.0070	0.0009
*TPTE*	NM_199261	13	0.0118	0.0095	1.2398
*CTAGE5*	NM_203356	14	0.0029	0.0082	0.3578
*BC048128*	BC048128	15	0.0077	0.0143	0.5355
*CASC5*	NM_170589	15	0.0084	0.0116	0.7226
*CCDC33*	NM_182791	15	0.0093	0.0192	0.4835
*Klkbl4*	XM_375358	16	0.0051	0.0109	0.4713
*HSPB9*	NM_033194	17	0.0112	0.0184	0.6077
*CTAGE1*	NM_172241	18	0.0108	0.0204	0.5311
*COX6B2*	NM_144613	19	0.0047	0.0138	0.3413
*DKKL1*	NM_014419	19	0.0055	0.0060	0.9034
*NALP4*	NM_134444	19	0.0090	0.0180	0.4981
*THEG*	NM_016585	19	0.0100	0.0091	1.1002
*TULP2*	NM_003323	19	0.0059	0.0056	1.0501
*CTCFL*	NM_080618	20	0.0124	0.0169	0.7316
*SPINLW1*	NM_181502	20	0.0134	0.0262	0.5122
*SPO11*	NM_012444	20	0.0044	0.0119	0.3679
*PRAME*	NM_006115	22	0.0191	0.0162	1.1798
*CPXCR1*	NM_033048	X	0.0104	0.0047	2.2411
*CSAG1*	NM_153478	X	0.0622	0.0006	∞
*CSAG2*	NM_004909	X	0.0163	0.0266	0.6138
*CT45-2*	NM_152582	X	0.0207	0.0002	∞
*DDX53*	NM_182699	X	0.0159	0.0109	1.4567
*FATE1*	NM_033085	X	0.0025	0.0142	0.1755
*FMR1NB*	NM_152578	X	0.0374	0.0228	1.6405
*FTHL17*	NM_031894	X	0.0150	0.0002	∞
*GAGE4*	NM_001474	X	0.0273	0.0117	2.3392
*GAGE8*	NM_012196	X	0.0244	0.0320	0.7617
*LUZP4*	NM_016383	X	0.0129	0.0138	0.9364
*MAGEA10*	NM_001011543	X	0.0083	0.0058	1.4380
*MAGEA11*	NM_001011544	X	0.0050	0.0055	0.9233
*MAGEA12*	NM_005367	X	0.0057	0.0222	0.2586
*MAGEA2*	NM_175743	X	0.0133	0.0126	1.0583
*MAGEA4*	NM_002362	X	0.0129	0.0086	1.4989
*MAGEA5*	NM_021049	X	0.0119	0.0001	∞
*MAGEA8*	NM_005364	X	0.0045	0.0074	0.6156
*MAGEA9*	NM_005365	X	0.0131	0.0171	0.7667
*MAGEB1*	NM_002363	X	0.0085	0.0129	0.6585
*MAGEB2*	NM_002364	X	0.0189	0.0068	2.7789
*MAGEB3*	NM_002365	X	0.0124	0.0001	∞
*MAGEB4*	NM_002367	X	0.0070	0.0133	0.5249
*MAGEB5*	XM_293407	X	0.0098	0.0117	0.8398
*MAGEB6*	NM_173523	X	0.0229	0.0157	1.4654
*NXF2*	NM_017809	X	0.0111	0.0125	0.8884
*PAGE1*	NM_003785	X	0.0102	0.0001	∞
*PAGE2B*	NM_001015038	X	0.0379	0.0117	3.2472
*PAGE3*	NM_001017931	X	0.0092	0.0087	1.0551
*PAGE4*	NM_007003	X	0.0000	0.0000	0.4989
*PAGE5*	NM_130467	X	0.0124	0.0001	∞
*SAGE1*	NM_018666	X	0.0096	0.0083	1.1487
*SPANX-N2*	NM_001009615	X	0.0216	0.0265	0.8131
*SPANX-N4*	NM_001009613	X	0.0151	0.0207	0.7276
*SPANX-N5*	NM_001009616	X	0.0000	0.0000	0.3869
*SPANXD*	NM_032417	X	0.1423	0.1107	1.2849
*SSX1*	NM_005635	X	0.0211	0.0057	3.7126
*SSX2*	NM_003147	X	0.0456	0.0373	1.2216
*SSX4*	NM_005636	X	0.0180	0.0059	3.0628
*SSX5*	NM_021015	X	0.0681	0.0622	1.0946
*SSX8*	NM_174961	X	0.0182	0.0002	∞
*SSX9*	NM_174962	X	0.0248	0.0208	1.1926
*XAGE1*	NM_133431	X	0.0145	0.0079	1.8487
*XAGE2*	NM_130777	X	0.0079	0.0001	∞
*XAGE3*	NM_133179	X	0.0046	0.0179	0.2556
*XAGE5*	NM_130775	X	0.0085	0.0118	0.7165
*TSPY1*	NM_003308	Y	0.0158	0.0241	0.6575

Synonymous (dS) and nonsynonymous (dN) nucleotide substitution rates were estimated using codeml from PAML [24] as described in Methods. Genes are presented by chromosomal location. '∞' denotes cases in which the dN/dS ratio cannot be calculated because the number of synonymous substitutions between the human and chimp sequences is zero.

**Table 3:** Significance of the differences in the distributions of dN/dS ratios between CT and control ORFs

**Comparison**	**p-value**
All CTs vs. All controls	6.22e-12
CT-Xs vs. Control-Xs	2.31e-10
Non-X CTs vs. Non-X controls	1.50e-05
CT-Xs vs. Non-X CTs	1.62e-05
Controls on X vs. Non-X controls	0.50

The distributions of dN/dS ratios from groups of CT and control ORFs were compared with each other, and any difference assessed using the non-parametric Mann-Whitney rank sum test [43]. Ratios denoted by '∞' in Table 2 were omitted from this analysis. For comparison, differences in the distributions were also assessed for significance using a parametric Welch two sample t-test; see additional file 3.

**Table 4:** Reports of positive selection pressure on CT genes

**CT_family**	**Gene name**	**Human RefSeq**	**Reference**	**Present work#**
CT1	*MAGEA4*	NM_002362	I	Yes
CT1	*MAGEA5*	NM_021049	I	Yes
CT1	*MAGEA10*	NM_021048	I	Yes
CT2	*BAGE2*	NM_182482	I	
CT3	*MAGEB2*	NM_002364	I	Yes
CT3	*MAGEB3*	NM_002365	I	Yes
CT5	*SSX1*	NM_005635	I, III	Yes
CT5	*SSX8*	NM_174961	I, III	Yes
CT7	*MAGEC2*	NM_016249	I	
CT7	*MAGEC3*	NM_138702	I	
CT11	*SPANX-N2*	NM_001009615	III	
CT11	*SPANX-N3*	NM_001009609	III	
CT11	*SPANX-N4*	NM_001009613	III	
CT11	*SPANX-N5*	NM_001009616	III	
CT11	*SPANXA*	NM_013453	III	
CT11	*SPANXB*	NM_032461	III	
CT11	*SPANXC*	NM_022661	III	
CT14	*SAGE1*	NM_018666	I, II	Yes
CT16	*PAGE1*	NM_003785	I	Yes
CT37	*FMR1NB*	NM_152578	I	Yes
CT38	*FTHL17*	NM_031894	I	Yes
CT48	*SLCO6A1*	NM_173488	I	
CT55	*CXorf48*	NM_017863	I	
CT56	*THEG*	NM_016585	I	Yes
CT63	*PASD1*	NM_173493	I	
CT65	*TULP2*	NM_003323	I	Yes
CT77	*CPXCR1*	NM_033048	I	Yes
CT80	*PIWIL2*	NM_018068	I	
CTNA	*PRAME*	NM_006115	I	Yes
CTNA	*PRAME*	cluster on chromosome 1	IV	Yes

Positive selection pressure on CT genes, from analysis of human and chimpanzee sequences, reported in: I, as defined by dN/dS > 1 [18, 33]. II, as defined by likelihood ratio test with p-value < 0.05 [35]. III, as defined by dN/dS > 1 [27] IV, inferred from dN/dS > 1 and sites modelling on human alignments [28] # Confirmed 16 previously reported positively selected CT genes, plus an additional 18 positively selected CT genes (see Table 2).
