# Supplementary material for: Rapid evolution of cancer/testis genes on the X chromosome
Source: BMC Genomics. 2007 May 23;8:129. doi: 10.1186/1471-2164-8-129 (PMC1890293; doi:10.1186/1471-2164-8-129)
Supplement: Additional File 3 — Significance of the differences in the distributions of dN/dS ratios between CT and control ORFs using a parametric t-test. Distribution of dN/dS ratios assessed by parametric t-test. The results are qualitatively similar to those presented in Table 3 and confirm that the distribution of dN/dS values is different between CT genes and controls. [file 1471-2164-8-129-S3.doc]

**Additional file 3. Significance of the differences in the distributions of dN/dS ratios between CT and control ORFs using a parametric t-test**

| **Comparison** | **Means** | **p-value** |
| --- | --- | --- |
| All CTs vs. All controls | 0.858, 0.365 | 6.00e-08 |
| CT-Xs vs. Control-Xs | 1.283, 0.329 | 9.02e-07 |
| Non-X CTs vs. Non-X controls | 0.571, 0.333 | 5.97e-04 |
| CT-Xs vs. Non-X CTs | 1.283, 0.571 | 9.42e-05 |
| Controls on X vs. Non-X controls | 0.329, 0.333 | 0.96 |

The distributions of dN/dS ratios from groups of CT and control ORFs were compared with each other, and any difference assessed using a parametric Welch two sample t-test [43]. Ratios denoted by ‘∞’ in Table 2 were omitted from this analysis. The mean of each distribution is shown in column two (same order as in column one).
